# Dopaminergic gene–epigenetic interactions and impulsivity traits in elite combat sport athletes

**DOI:** 10.3389/fpsyg.2026.1807306

**Published:** 2026-06-01

**Authors:** Remigiusz Recław, Jolanta Chmielowiec, Krzysztof Chmielowiec, Jolanta Masiak, Anna Grzywacz

**Affiliations:** 1 Independent Laboratory of Genetics and Behavioral Epigenetics, Pomeranian Medical University in Szczecin, Szczecin, Poland; 2 Department of Medical Sciences and Public Health, Gdańsk University of Physical Education and Sport, Gdańsk, Poland; 3 Department of Nursing, Collegium Medicum, University of Zielona Góra, Zielona Góra, Poland; 4 Department of Hygiene and Epidemiology, Collegium Medicum, University of Zielona Góra, Zielona Góra, Poland; 5 Second Department of Psychiatry and Psychiatric Rehabilitation, Medical University of Lublin, Lublin, Poland

**Keywords:** *DAT1* (SLC6A3) promoter methylation, *DRD2* rs1076560, elite combat sports, gene–epigenome interaction, impulsivity, self-regulation

## Abstract

Dopaminergic signaling is a key mechanism underlying behavioral regulation and impulse control. While epigenetic variation in dopaminergic genes has been linked to dysregulated behavior in clinical populations, its relevance in high-functioning individuals remains less clear. Elite combat sport athletes provide an informative model for studying self-control in performance-demanding environments. This study examined differences between combat sport athletes and matched controls in *DAT1* (SLC6A3) promoter methylation, *DRD2* rs1076560 genotype, and trait impulsivity, and tested whether *DRD2* variation moderates methylation patterns. A total of 209 males (*n* = 100 athletes; *n* = 109 controls) were included. Methylation across 33 CpG sites in the *DAT1* promoter was quantified from whole-blood DNA, *DRD2* rs1076560 was genotyped using real-time PCR, and impulsivity was assessed with the Barratt Impulsiveness Scale (BIS-11). Athletes showed higher *DAT1* methylation (*Z* = 7.022, *p* < 0.0001) and lower impulsivity across BIS-11 domains (all *p* < 0.01) than controls. A significant group × genotype interaction was observed for *DAT1* methylation (*F*_1,204_ = 14.05, *p* = 0.0002), with A allele carriers showing the highest methylation among athletes. No direct genotype effect on impulsivity was found. These results suggest experience-related dopaminergic epigenetic differences among elite athletes, potentially shaped by genetic background, and support integrative models of self-regulation in high-performance contexts. Such biomarkers may help inform individualized approaches to psychological training and performance resilience.

## 1 Introduction

Combat sports, such as mixed martial arts, boxing, judo, and karate, require exceptional physical, cognitive, and emotional skills. Success depends not only on strength and endurance, but also on advanced emotional regulation, rapid decision-making, and impulse control. Therefore, combat sport athletes constitute a valuable model for exploring biological and psychological mechanisms of self-regulation in healthy, high-functioning individuals exposed to high-stress environments (Woodman and Hardy, 2003). Understanding the biological substrates of self-regulation in elite combat athletes may provide insight into mechanisms supporting psychological resilience and long-term wellbeing in high-demand sport environments. In our previous work (Recław et al., 2025), we suggested that *DAT1* promoter methylation may reflect experience-dependent biological adaptation in elite combat sport athletes. In that analysis, we focused on the VNTR polymorphism in the 3′-untranslated region (3′-UTR) of the *DAT1* (SLC6A3) gene, which primarily influences mRNA stability, transporter expression, and dopamine reuptake efficiency. In the present study, we conducted a secondary analysis of the same cohort to address a distinct mechanistic research question, namely whether *DAT1* promoter methylation interacts with the *DRD2* rs1076560 polymorphism in relation to impulsivity-related traits. This approach extends our earlier findings by shifting from general group-level epigenetic differences to a genotype-moderated model of individual variability in behavioral self-regulation.

Integrating genetic, epigenetic, and psychological approaches offers a comprehensive view of individual variability in traits relevant to sports performance (Pickering and Kiely, 2018; Pitsiladis et al., 2013). In recent years, sports genetics research has increasingly emphasized biologically rooted individual differences in performance and behavioral regulation, highlighting the value of integrative approaches that combine genetic, epigenetic, and psychological perspectives in elite athlete populations (Pak et al., 2021; Wiers et al., 2018).

Genetic polymorphisms may influence neurotransmitter signaling, stress reactivity, and decision-making processes, while epigenetic mechanisms such as DNA methylation may reflect longer-term adaptation to environmental demands. Psychological assessments capture the behavioral expression of these biological processes. However, few studies have simultaneously examined genetic and epigenetic factors in relation to impulsivity-related cognitive–emotional traits in elite sport populations (Varillas-Delgado et al., 2022). In particular, the contribution of dopaminergic gene–epigenome interactions to individual differences in self-control in elite athletes remains insufficiently understood.

The dopaminergic system plays a central role in reward processing, goal-directed behavior, and impulse inhibition (Volkow and Wise, 2005). The *DRD2* gene encodes the dopamine D2 receptor, and the intronic SNP rs1076560 affects alternative splicing into presynaptic (D2S) and postsynaptic (D2L) isoforms. The A allele has been associated with reduced D2S expression, shifting signaling toward the postsynaptic pathway, and has been linked to impulsivity, risk-taking, and altered reward-related brain activity (Zhang et al., 2007; Clarke et al., 2014; Moyer et al., 2011; Persico and Napolioni, 2013; Luykx et al., 2017). These functional effects differ mechanistically from those of the *DAT1* VNTR, which acts predominantly at the level of transporter expression and availability rather than receptor isoform balance – thereby justifying a separate examination of its interaction with *DAT1* promoter methylation in the context of training-induced neuroadaptations in elite athletes.

Another regulator of dopaminergic tone is the dopamine transporter (*DAT1*, also known as SLC6A3), responsible for synaptic dopamine reuptake. Promoter-region methylation may downregulate *DAT1* expression, influencing dopamine availability. Altered *DAT1* methylation has been associated with ADHD and substance use disorders (Walton et al., 2016). However, its role in impulsivity-related behavioral traits in healthy, high-performing individuals remains underexplored (Recław et al., 2024).

This study examined the interaction between *DRD2* rs1076560 and *DAT1* promoter methylation in relation to impulsivity among combat sport athletes and matched controls. Impulsivity was assessed using the Barratt Impulsiveness Scale–Version 11 (BIS-11), which measures attentional, motor, and non-planning dimensions (Patton et al., 1995). We hypothesized that *DAT1* promoter methylation would show group-dependent associations with impulsivity, and that these effects would be moderated by *DRD2* rs1076560, reflecting dopaminergic pathway-level gene-epigenome interplay.

## 2 Materials and methods

### 2.1 Participants

The study sample comprised 100 male combat sport athletes (mean age = 21.96; SD = 4.61) and a control group of 109 healthy male volunteers matched for age (mean age = 21.86; SD = 3.85). Prior to participation, all individuals were informed about the purpose of the study and provided written informed consent. To minimize potential bias related to genetic variability across populations, both groups included individuals of Caucasian origin from the same geographical region in Poland.

The athlete group consisted of healthy Polish males with no reported history of substance dependence or psychotic disorders. Participants were engaged in various combat sports disciplines: mixed martial arts (MMA, *n* = 37), judo (*n* = 22), boxing (*n* = 19), karate (*n* = 14), and kickboxing (*n* = 8). Recruitment was carried out through multiple channels, including cooperation with national sport teams and outreach to coaches and athletes during training camps.

### 2.2 Ethics statement

The research protocol adhered to the ethical standards outlined in the Declaration of Helsinki and received approval from the Bioethics Committee of the Regional Medical Chamber in Szczecin (protocol no. 13/KB/VI/2016, December 8, 2016).

### 2.3 Measures

Impulsivity was assessed using the Barratt Impulsiveness Scale, 11th Edition (BIS-11), a 30-item self-report questionnaire measuring trait-level impulsivity (Chmielowiec et al., 2023). The BIS-11 captures three core dimensions: attentional impulsivity (inattention and cognitive instability), motor impulsivity (acting without thinking), and non-planning impulsivity (reduced future orientation and forethought). Items are rated on a 4-point Likert scale from “rarely/never” to “almost always/always,” with higher scores indicating greater impulsivity. Items requiring reverse scoring were recoded according to the standard BIS-11 scoring procedure prior to calculating subscale and total scores. Subscale scores and the total BIS-11 score were calculated and used in subsequent analyses. The BIS-11 is widely applied in both clinical and non-clinical research as an indicator of individual differences in self-regulation and impulsivity-related behavioral tendencies. In the context of elite sport, BIS-11 provides a standardized measure of impulsivity-related traits relevant to behavioral control under high-pressure conditions.

The internal consistency of the BIS-11 scale was assessed using Cronbach’s alpha. In the analyzed sample (*N* = 209), the Cronbach’s alpha coefficient for the total scale score was *α* = 0.78 (standardized α = 0.77), indicating good internal consistency of the instrument. Cronbach’s alpha values for the individual subscales were as follows: attentional impulsivity (BIS-AI) α = 0.66, motor impulsivity (BIS-MI) α = 0.70, non-planning impulsivity (BIS-NI) α = 0.76. The mean inter-item correlation was *r* = 0.55. Correlations between the subscales and the total score were high, with *r* = 0.67 for BIS-AI, *r* = 0.63 for BIS-MI, and *r* = 0.57 for BIS-NI.

### 2.4 Genotyping and DNA methylation

Peripheral blood samples were collected from both groups via venipuncture and used for genomic DNA isolation. The extraction was carried out using a modified salting-out method, a standard procedure in molecular genetics. The concentration and purity of the DNA were verified using spectrophotometric analysis. Following quality control, all samples were stored at −20 °C pending further procedures.

DNA methylation was assessed following bisulfite conversion of 250 ng genomic DNA using the EZ DNA Methylation Kit (Zymo Research, Orange, CA, United States), strictly adhering to the manufacturer’s protocol. A bisulfite-specific PCR was performed on a Mastercycler epgradient S thermal cycler (Eppendorf, Germany) to amplify a 447 bp fragment of the *DAT1* gene promoter (ENSG00000142319) encompassing 33 CpG sites. Primers were designed using MethPrimer (http://www.urogene.org/cgi-bin/methprimer/methprimer.cgi, accessed 29 April 2022) and synthesized by Genomed.pl. (Warsaw, Poland): forward DATF 5′-GGTTTTTGTTTTTTTTATTGTTGAG-3′, reverse DATR 5′-AAATCCCCTAAACCTAATCCC-3′.

PCR products were sequenced using the BigDye Terminator v3.1 Cycle Sequencing Kit (Applied Biosystems, Foster City, CA, United States) on an ABI Prism 3130XL Genetic Analyzer. Chromatograms were analyzed in 4Peaks software (Nucleobytes, Aalsmeer, The Netherlands). For each CpG site, methylation was quantified as G/(G + A) × 100%; sites with a signal ≥20% were classified as methylated. The primary epigenetic outcome was the total number of methylated CpG sites (0–33) per sample, following the validated protocol (Recław et al., 2024). Conversion efficiency was verified by inspecting non-CpG cytosines, which showed ≥98% conversion, and no-template controls (NTC) were included in every PCR run to detect potential contamination.

In addition, genotyping of the *DRD2* rs1076560 (C > A) single nucleotide polymorphism (SNP) was performed using real-time PCR with melting curve analysis. The amplification employed allele-specific fluorescent probes and was run on the LightCycler^®^ 480 platform (Roche Diagnostics, Mannheim, Germany), following manufacturer-supplied guidelines.

Melting curve analysis allowed for genotype discrimination based on characteristic melting temperatures (Tm), with distinct peaks detected at approximately 56.6 °C for the C allele and 62.9 °C for the A allele. Each reaction included internal quality controls, both positive and negative, and to ensure reproducibility, 10% of randomly selected samples were re-analyzed in duplicate, resulting in a concordance rate exceeding 99%. The detailed methodology for SNP genotyping has been thoroughly described in earlier publications from our research team.

### 2.5 Statistical analysis

Hardy–Weinberg equilibrium (HWE) for genotype frequencies was assessed using the dedicated calculator available at https://wpcalc.com/en/equilibrium-hardy-weinberg/ (accessed on 12 December 2024). To examine the effects of group (combat sport athletes vs. controls) and genotype (*DRD2* rs1076560) on impulsivity and methylation, a factorial 2 (group) × 3 (genotype) analysis of variance (ANOVA) was conducted separately for each dependent variable: BIS-11 subscales and the number of methylated CpG sites in the *DAT1* gene.

The assumptions for ANOVA were tested: the homogeneity of variances was confirmed using Levene’s test (*p* > 0.05), although data did not meet the normality assumption. Therefore, additional group comparisons (combat athletes vs. controls) for the BIS-11 subscales and *DAT1* methylation were performed using the non-parametric Mann–Whitney U test. The use of both ANOVA and Mann–Whitney U was intended to ensure robustness of results, allowing for factorial analysis of interaction effects despite deviations from normality, while also validating pairwise group differences with a distribution-free method.

Chi-square tests (*χ*^2^) were used to compare the distribution of *DRD2* rs1076560 genotypes and alleles between the groups. All statistical procedures were carried out using STATISTICA 13 (Tibco Software Inc., Palo Alto, CA, United States) for Windows (Microsoft Corporation, Redmond, WA, United States). A significance level of *α* = 0.05 was applied for all analyses. For the variables related to BIS-11 subscale scores and *DAT1* gene methylation site counts, a significance level of α = 0.01 (0.05/5) was adopted, using a Bonferroni correction for multiple comparisons. A *post hoc* power analysis was conducted for the main interaction effect (group × genotype) in the factorial ANOVA model, indicating high statistical power (power = 0.96).

## 3 Results

The genotype distribution in both combat sport athletes and control subjects was consistent with Hardy–Weinberg equilibrium assumptions (*p* > 0.05 in both groups; see Table 1).

**Table 1:** Genotype distribution and Hardy–Weinberg equilibrium in combat sport athletes and control subjects.

Hardy–Weinberg equilibrium calculator including analysis for ascertainment bias		Observed (expected)	Allele freq	*χ*^2^ (*p*-value)
*DRD2* rs1076560				
Combat sport athletes *n* = 100	C/C	76 (74.8)	p (C) = 0.86 q (A) = 0.14	1.017 (0.3133)
	A/C	21 (23.4)		
	A/A	3 (1.8)		
Control *n* = 109	C/C	76 (73.5)	p (C) = 0.82 q (A) = 0.18	2.682 (0.1015)
	A/C	27 (32.0)		
	A/A	6 (3.5)		

*p*, statistical significance *χ*^2^ test.

No significant differences were found in the distribution of *DRD2* rs1076560 genotypes between combat sport athletes and the control group. The observed frequencies were as follows: C/C – 0.76 vs. 0.70; A/C – 0.21 vs. 0.25; A/A – 0.03 vs. 0.06 (χ^2^ = 1.365, *p* = 0.5053). Similarly, the comparison of allele frequencies showed no statistical difference between groups: C – 0.86 vs. 0.82; A – 0.14 vs. 0.18 (χ^2^ = 1.512, *p* = 0.2189; see Table 2).

**Table 2:** Frequency of *DRD2* rs1076560 genotypes and alleles in combat sport athletes and control subjects.

*DRD2* rs1076560					
	Genotypes			Alleles	
	C/C *n* (%)	A/C *n* (%)	A/A *n* (%)	C *n* (%)	A *n* (%)
Combat sport athletes *n* = 100	76 (76.00%)	21 (21.00%)	3 (3.00%)	173 (86.50%)	27 (13.50%)
Control *n* = 109	76 (69.73%)	27 (24.77%)	6 (5.50%)	179 (82.11%)	39 (17.89%)
*χ*^2^ (*p*-value)	1.365 0.5053			1.512 (0.2189)	

*n*, number of subjects.

Table 3 presents the means and standard deviations for all BIS subscales, as well as the number of methylated sites within the *DAT1* gene, separately for combat sport athletes and control participants.

**Table 3:** BIS-11 scores and number of methylated sites in the DAT1 gene in combat sport athletes and control subjects.

BIS-11 Number of methylation sites in the *DAT1* gene	Combat sport athletes *n* = 100	Controls *n* = 109	*Z*	(*p*-value)
BIS-AI	16.53 ± 3.45	17.83 ± 3.72	−2.468	0.0136*
BIS-MI	21.76 ± 4.26	23.86 ± 4.06	−3.763	0.0002#
BIS-NI	25.31 ± 4.03	27.53 ± 3.77	−4.131	0.0001#
BIS-Total	63.46 ± 9.83	69.05 ± 9.53	−4.008	0.0001#
Number of methylation sites in the *DAT1* gene	17.42 ± 6.40	11.74 ± 3.80	7.022	0.0001#

*p*, statistical significance with Mann–Whitney *U*-test; *n*, number of subjects; M ± SD, mean ± standard deviation; *, statistically significant differences; #, Bonferroni correction: the *p*-value threshold was lowered to 0.01 (0.05/5 = 0.01).

Combat sport athletes demonstrated a significantly greater number of methylated sites within the *DAT1* gene compared to the control group (17.42 vs. 11.74; Z = 7.022; *p* < 0.0001; see Table 3). In contrast, their impulsivity scores were consistently lower across all subscales of the Barratt Impulsiveness Scale (BIS). Specifically, combat athletes scored lower than controls on the BIS-AI (16.53 vs. 17.83; *Z* = −2.468; *p* = 0.0136), the BIS-MI (21.76 vs. 23.86; *Z* = −3.763; *p* = 0.0002), the BIS-NI (25.31 vs. 27.53; *Z* = −4.131; *p* < 0.0001), and the BIS-Total (63.46 vs. 69.05; *Z* = −4.008; *p* < 0.0001; see Table 3). Importantly, in each of these comparisons, the differences reached statistical significance, indicating robust group differences.

Results from the 2 × 3 factorial ANOVA examining BIS-11 scores and the number of methylated *DAT1* gene sites are summarized in Table 4.

**Table 4:** Differences in *DRD2* rs1076560 genotype, BIS-11 scores, and number of methylated sites in the *DAT1* gene between combat sport athletes (CSA) and control subjects.

BIS-11	Group	*DRD2* rs1076560		Factor	ANOVA		
		C/C *n* = 152 *M* ± SD	A/C + A/A *n* = 57 *M* ± SD		*F* (*p*-value)	*ɳ* ^2^	Power (alpha = 0.05)
BIS-AI	CSA; *n* = 100	16.59 ± 3.65	16.33 ± 2.79	Intercept CSA /control *DRD2* CSA /control x *DRD2*	*F*_1,204_ = 3674.81 (*p* < 0.0001)*#	0.947	1.000
	Control; *n* = 109	17.89 ± 4.05	17.68 ± 2.93		*F*_1,204_ = 5.50 (*p =* 0.0199)*	0.026	0.646
					*F*_1,204_ = 0.17 (*p* = 0.677)	0.001	0.070
					*F*_1,204_ = 0.01 (*p* = 0.9742)	<0.001	0.050
BIS-MI	CSA; *n* = 100	21.92 ± 4.52	21.25 ± 3.33	Intercept CSA /control *DRD2* CSA /control x *DRD2*	*F*_1,204_ = 4880.77 (*p* < 0.0001)*#	0.960	1.000
	Control; *n* = 109	23.73 ± 4.19	24.15 ± 3.81		*F*_1,204_ = 13.06 (*p =* 0.0004)*#	0.060	0.949
					*F*_1,204_ = 0.04 (*p* = 0.8443)	0.0002	0.054
					*F*_1,204_ = 0.69 (*p* = 0.4066)	0.003	0.131
BIS-NI	CSA; *n* = 100	25.39 ± 4.13	25.08 ± 3.79	Intercept CSA /control *DRD2* CSA /control x *DRD2*	*F*_1,204_ = 7452.43 (*p* < 0.0001)*#	0.973	1.000
	Control; *n* = 109	27.47 ± 3.65	27.68 ± 4.07		*F*_1,204_ = 14.59 (*p =* 0.0002)*#	0.067	0.967
					*F*_1,204_ = 0.01 (*p* = 0.9391)	<0.001	0.051
					*F*_1,204_ = 0.18 (*p* = 0.6753)	0.001	0.070
BIS-Total	CSA; *n* = 100	63.72 ± 10.45	62.67 ± 7.72	Intercept CSA /control *DRD2* CSA /control x *DRD2*	*F*_1,204_ = 7595.23 (*p* < 0.0001)*#	0.974	1.000
	Control; *n* = 109	68.84 ± 9.89	69.50 ± 8.82				
					*F*_1,204_ = 15.49 (*p =* 0.0001)*#	0.071	0.975
					*F*_1,204_ = 0.02 (*p* = 0.8971)	<0.001	0.052
					*F*_1,204_ = 0.32 (*p* = 0.5733)	0.002	0.087
Number of methylation sites in the *DAT1* gene	CSA; *n* = 100	16.21 ± 6.38	21.25 ± 4.83	Intercept CSA /control *DRD2* CSA /control x *DRD2*	*F*_1,204_ = 1508.54 (*p* < 0.0001)*#	0.880	1.000
	Control; n = 109	11.99 ± 3.81	11.18 ± 3.79		*F*_1,204_ = 83.90 (*p <* 0.0001)*#	0.289	1.000
					*F*_1,204_ = 7.34 (*p* = 0.0073)*#	0.034	0.769
					*F*_1,204_ = 14.05 (*p* = 0.0002)*#	0.064	0.962

*, significant result; CSA, combat sport athletes; *M* ± SD, mean ± standard deviation; *n*, number of subjects; *p*, statistical significance (ANOVA test); *η*^2^, effect size (partial eta squared); #, Bonferroni correction: the *p*-value threshold was lowered to 0.01 (0.05/5 = 0.01).

**Figure 1:**
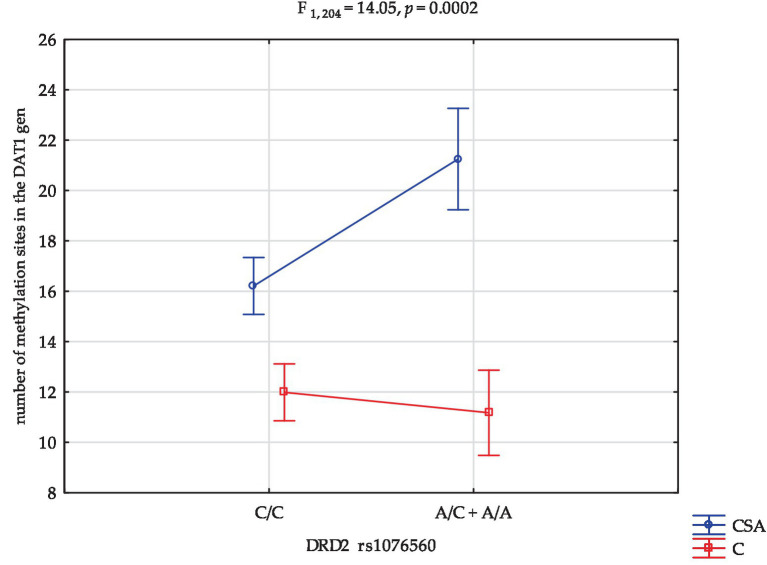
Interaction between group (combat sport athletes vs. controls) and *DRD2* rs1076560 genotype on the number of methylated sites in the *DAT1* gene.

A significant interaction effect was observed between *DRD2* rs1076560 genotype and group (combat sport athletes vs. controls) on the number of methylated *DAT1* gene sites (*F*_1,204_ = 14.05; *p* = 0.0002; *η*^2^ = 0.064; see Table 4 and Figure 1). The statistical power for this factor was estimated at 96%, with approximately 6% of the variance in methylation levels explained by the interaction between *DRD2* rs1076560 genotype and group affiliation.

Detailed *post hoc* comparisons are provided in Table 5. These results revealed that combat sport athletes (CSA) with the C/C genotype exhibited significantly fewer methylated *DAT1* sites compared to their counterparts with A/C + A/A genotypes. However, CSA individuals with the C/C genotype still showed significantly higher methylation levels than control participants (C) with either C/C or A/C + A/A genotypes. Furthermore, CSA participants with A/C + A/A genotypes had significantly higher numbers of methylation sites than control subjects with genotypes C/C and A/C + A/A.

**Table 5:** *Post hoc* least significant difference analysis of the interaction between *DRD2* rs1076560 genotype and group (combat sport athletes vs. controls) on the number of methylated sites in the *DAT1* gene.

*DRD2* rs1076560 and number of methylation sites in the *DAT1* gen				
	{1} *M* = 16.21	{2} *M* = 21.25	{3} *M* = 11.99	{4} *M* = 11.18
Combat sport athletes C/C {1}		<0.0001*	<0.0001*	<0.0001*
Combat sport athletes A/C + A/A{2}			<0.0001*	<0.0001
Control C/C {3}				0.4332
Control A/C + A/A{4}				

CSA, combat sport athletes; *, significant statistical differences, M, mean.

## 4 Discussion

### 4.1 Summary of main findings

This study revealed clear group-level differences in dopaminergic epigenetic markers and trait impulsivity, as well as a significant group × genotype interaction for *DAT1* promoter methylation. Athletes displayed higher *DAT1* methylation than controls, a pattern that may be consistent with experience-related differences in dopaminergic regulation associated with long-term participation in elite combat sports (Chmielowiec et al., 2023; Wiers et al., 2018; Pak et al., 2021; Voisin et al., 2015). Importantly, *DAT1* methylation was moderated by *DRD2* rs1076560 genotype, in line with previous evidence linking this variant to altered *DRD2* splicing and dopaminergic signaling (Zhang et al., 2007; Clarke et al., 2014; Moyer et al., 2011). At the behavioral level, combat sport athletes reported lower impulsivity across BIS-11 domains compared to controls, supporting the interpretation that elite combat sport athletes may exhibit enhanced self-regulation.

### 4.2 Biological interpretation of *DAT1* methylation

Elevated *DAT1* promoter methylation observed in combat sport athletes may reflect experience-related biological differences associated with sustained high-performance training. Methylation within the 5′ regulatory region of *DAT1* (SLC6A3) has been linked to reduced gene expression and lower dopamine transporter (*DAT*) availability, potentially resulting in slower dopamine reuptake and prolonged dopaminergic signaling (Chmielowiec et al., 2023; Wiers et al., 2018). However, it should be noted that the present study did not directly measure *DAT1* gene expression. Therefore, the proposed relationship between promoter methylation and transporter availability should be interpreted cautiously and viewed as a hypothesis consistent with previous studies rather than a direct functional conclusion derived from the present data. Such dopaminergic tuning may be relevant for processes such as motivational salience, reward responsivity, and sustained attentional engagement, which are crucial for competitive performance (Pak et al., 2021).

From an environmental perspective, chronic exposure to performance pressure, repeated goal-directed behaviors, and intensive emotion regulation demands may contribute to epigenetic plasticity within dopaminergic pathways (Voisin et al., 2015; Denham, 2017). In this context, athletic training can be conceptualized as a form of structured environmental enrichment that may shape regulatory biological profiles over time.

Notably, higher *DAT1* methylation in athletes contrasts with findings reported in clinical populations characterized by impaired impulse control (e.g., ADHD or stimulant addiction) (Recław et al., 2024). However, comparisons across studies should be made cautiously due to differences in tissues, CpG coverage, and quantification methods. Moreover, methylation markers measured in peripheral blood provide only indirect insight into central nervous system mechanisms and should be interpreted as correlates rather than definitive functional readouts.

Finally, the observed pattern is consistent with systems-level models in which dopamine tone is shaped by the interplay between genetic background and environmental exposure, potentially enabling context-dependent optimization of behavioral regulation (Chmielowiec et al., 2023; Green et al., 2015).

### 4.3 Role of *DRD2* rs1076560 and its interaction with *DAT1* methylation

The *DRD2* rs1076560 polymorphism has been extensively examined in relation to dopaminergic signaling and individual differences in behavioral regulation. This intronic SNP influences alternative splicing of the *DRD2* transcript, thereby affecting the balance between the D2S (presynaptic) and D2L (postsynaptic) receptor isoforms. The A allele has been associated with reduced D2S expression and a relative shift toward D2L, which may alter dopamine autoregulatory feedback and enhance postsynaptic signaling (Zhang et al., 2007; Bertolino et al., 2009). Such functional differences have been linked to impulsivity-related phenotypes, reward sensitivity, and vulnerability to psychiatric conditions in some studies, including addiction and schizophrenia (Moyer et al., 2011; Cohen et al., 2016).

In the present study, we did not detect a direct main effect of *DRD2* rs1076560 genotype on BIS-11 impulsivity scores. Although this may appear inconsistent with earlier reports, it is also in line with evidence that single genetic variants often show small, context-dependent behavioral effects, particularly in non-clinical samples where trait variability is relatively constrained and compensatory processes may operate (Blasi et al., 2009). Thus, the absence of a direct genotype–behavior association does not necessarily contradict prior findings but may instead highlight the importance of intermediate biological mechanisms.

Importantly, we observed a significant interaction between *DRD2* genotype and group status (athletes vs. controls) in predicting *DAT1* promoter methylation. Specifically, among athletes, A allele carriers (A/C and A/A) exhibited substantially higher *DAT1* methylation levels than C/C homozygotes, whereas this pattern was not observed in the control group. This suggests that DRD2-related dopaminergic variability may shape experience-dependent epigenetic profiles, particularly under conditions characterized by sustained environmental demands, such as elite combat sport training and repeated exposure to high-arousal performance contexts.

One plausible interpretation is that *DAT1* methylation may serve as a regulatory or compensatory mechanism buffering dopaminergic signaling in individuals carrying the A allele. Higher *DAT1* promoter methylation could be associated with reduced *DAT1* expression and lower dopamine reuptake, potentially counterbalancing altered receptor signaling dynamics by stabilizing dopamine tone – a pattern consistent with homeostatic adjustment within the dopaminergic system. Conceptually, these results align with a gene × environment × epigenome framework, in which genetic predispositions interact with intensive training exposure and chronic performance-related stress to shape epigenetic regulation, which may ultimately support adaptive self-regulation.

To our knowledge, this is the first study to report an interaction between *DRD2* rs1076560 genotype and *DAT1* methylation in a healthy, high-functioning athletic population. The findings highlight the value of investigating dopaminergic regulation in elite sport samples, where behavioral traits may reflect not only vulnerability pathways but also adaptive processes that remain less apparent in clinical cohorts.

### 4.4 A sport-psychology interpretation: self-regulation under high pressure

Elite combat sports create repeated exposure to high-arousal situations requiring rapid action selection and inhibition of maladaptive impulses (Woodman and Hardy, 2003). From a sport psychology perspective, lower BIS-11 scores may reflect not only reduced impulsive responding, but also stronger top–down self-regulation, attentional control, and strategic decision-making under pressure (Kalén et al., 2021). The observed elevation in *DAT1* promoter methylation among athletes may represent a biological correlate of this long-term behavioral calibration, potentially shaped by chronic training demands and competitive stress exposure. Importantly, these patterns likely reflect a combination of selection effects (individuals with higher baseline self-control remaining in elite sport) and training-related adaptation (plastic changes resulting from sustained practice). The significant *DRD2* × group interaction suggests that genetic background may modulate individual sensitivity to experience-dependent epigenetic regulation, supporting a gene × environment × epigenome framework of self-regulation in high-performance contexts (Recław et al., 2025).

### 4.5 Implications for sport psychology and performance

The biological patterns observed in this study – higher *DAT1* promoter methylation in combat sport athletes and its interaction with *DRD2* rs1076560 – suggest that intensive athletic engagement may be associated with experience-dependent neurobiological adaptation supporting self-regulation. Elevated methylation, particularly among A allele carriers, may reflect a regulatory adjustment within dopaminergic signaling under high-arousal and high-stress conditions typical of combat sports, potentially contributing to performance consistency, impulse control, and emotional resilience (Recław et al., 2024; Voisin et al., 2015).

The lower impulsivity profile observed in athletes is consistent with evidence that elite performance depends on refined cognitive-emotional control, including attentional focus, resilience, and the capacity to sustain goal-directed behavior under pressure (Denham, 2017; MacNamara and Collins, 2015). Taken together, the findings support integrative biopsychosocial perspectives in sport psychology, in which long-term training experience interacts with biological predispositions to shape behavioral regulation and performance-relevant psychological traits.

From an applied standpoint, these results may inform individualized mental skills training aimed at optimizing self-regulation in competitive environments. Interventions such as mindfulness-based approaches, attentional control routines, emotion regulation strategies, or neurofeedback may be particularly relevant for athletes who exhibit less favorable self-regulatory profiles or higher vulnerability to stress-related dysregulation. Additionally, *DAT1* promoter methylation could be explored as a candidate marker of adaptation to training load or chronic stress exposure, although replication and longitudinal validation are required before any practical implementation (Voisin et al., 2015; Dudek et al., 2015).

### 4.6 Comparison with existing literature

Altered *DAT1* promoter methylation has been reported in clinical conditions characterized by impaired behavioral regulation, including ADHD and substance use disorders (Walton et al., 2016; Recław et al., 2024). Such populations often show lower *DAT1* methylation, whereas the elevated methylation observed in the present athlete sample may reflect a different regulatory pattern potentially associated with adaptive functioning under sustained cognitive and emotional demands (Voisin et al., 2015). This interpretation is consistent with broader models of experience-dependent epigenetic plasticity, in which repeated environmental exposure can shape stable biological profiles relevant to self-regulation.

The *DRD2* rs1076560 A allele, linked to reduced presynaptic D2S expression and a relative shift toward postsynaptic D2L signaling, has been associated with substance abuse vulnerability, impulsivity-related phenotypes, and altered frontostriatal functioning in several studies (Zhang et al., 2007; Clarke et al., 2014; Moyer et al., 2011; Persico and Napolioni, 2013; Luykx et al., 2017). In contrast, we did not observe a direct genotype effect on BIS-11 impulsivity, which may reflect the modest and context-dependent nature of single-variant behavioral associations in non-clinical samples. Instead, our findings highlight an interaction pattern: among athletes, A allele carriers displayed higher *DAT1* promoter methylation, suggesting that intensive training exposure may interact with dopaminergic genetic background to shape epigenetic regulation.

Prior research on impulsivity in athletes remains mixed. Some studies report elevated impulsivity in disciplines characterized by high physical risk and sensation seeking (e.g., extreme sports) (Dudek et al., 2015), whereas others indicate enhanced executive control and self-regulatory capacity in elite performers (MacNamara and Collins, 2015). Importantly, trait impulsivity assessed via BIS-11 captures attentional, motor, and non-planning dimensions, which may not fully overlap with adaptive risk-taking or rapid decision-making that can be advantageous in certain sport contexts. Overall, our results support the view that combat sports may be associated with refined impulse regulation, accompanied by measurable dopaminergic epigenetic differences.

### 4.7 Limitations

Several limitations of this study should be acknowledged. First, the sample size, although sufficient for detecting medium to large effects in the main analyses, may have limited statistical power for detecting subtler genetic associations – particularly for rare genotypes. In the case of *DRD2* rs1076560, the frequency of the A/A genotype was relatively low, which could have masked potential direct effects on impulsivity or interactive effects with environmental exposure. Future studies with larger and more genetically balanced samples are needed to verify these findings.

Second, the study included only male participants. While this approach minimized potential confounding effects of sex-related differences in hormone profiles or epigenetic patterns, it limits the generalizability of the findings to female athletes. Future research should investigate whether similar gene–epigenome–behavior interactions occur in women, as sex hormones can influence both dopaminergic function and DNA methylation patterns.

Third, the use of DNA extracted from leukocytes in whole blood introduces a potential limitation regarding tissue specificity. Although peripheral methylation patterns have been shown to correlate moderately with brain tissue methylation in some genes, this relationship is not universal. Thus, caution is warranted when extrapolating our results to central nervous system mechanisms. Further studies using neural tissue or neuroimaging correlates of methylation are needed to strengthen biological inference. Consequently, the methylation patterns observed in this study should be interpreted as peripheral epigenetic markers potentially associated with systemic biological processes related to training exposure, rather than as direct indicators of central nervous system epigenetic regulation.

Fourth, the study’s cross-sectional design precludes causal conclusions. While we observed associations between elite combat sport participation, epigenetic regulation, and impulsivity, we cannot determine whether these methylation patterns are a cause or a consequence of sport engagement. Longitudinal designs would help clarify the temporal dynamics of gene–environment interactions.

Fifth, impulsivity was assessed solely through self-report (BIS-11), which may not capture the full behavioral complexity of the trait. Although BIS-11 is a validated and widely used tool, future work could benefit from multimodal assessment, including behavioral tasks, informant reports, and real-world performance data. Moreover, self-report measures may not fully capture inhibitory control expressed under acute competitive pressure.

Sixth, the present study represents a secondary analysis of a previously published cohort. Although the current work addresses a distinct mechanistic question focused on *DRD2*-related moderation of *DAT1* promoter methylation, replication in an independent sample is needed to confirm the robustness and generalizability of the observed interaction effects; therefore, the results should be interpreted cautiously until replicated.

Finally, although we focused on two key dopaminergic markers (*DAT1* and *DRD2*), dopaminergic regulation is polygenic and interacts with other neurotransmitter systems (e.g., serotonin, noradrenaline). A more comprehensive genetic and epigenetic profile could provide a richer understanding of the biological basis of self-regulation in athletes.

Therefore, the present findings should be interpreted within a dual-framework, in which both self-selection into elite sport and long-term sport-related exposure may contribute to the observed biological and psychological profile.

### 4.8 Future directions

Future research should explore longitudinal changes in gene–environment–epigenome interactions to determine whether the observed methylation patterns are a result of training adaptations or reflect pre-existing traits. Such designs would also clarify the degree of methylation plasticity in response to environmental shifts, consistent with evidence showing that life experiences can leave lasting epigenetic marks in various physiological systems, though this remains largely unexplored in the dopaminergic domain (Provencal and Binder, 2015).

Broadening the genetic scope is another priority. Besides *DAT1* and *DRD2*, genes such as *COMT*, *MAOA*, and *BDNF* may also influence self-regulation. Prior studies indicate that interaction effects between *DAT1* and *COMT* variants can moderate impulsivity levels, supporting the use of polygenic approaches to capture the complex genetic architecture of behavioral regulation (Paloyelis et al., 2010).

Combining genetic data with neuroimaging (e.g., fMRI, connectivity analyses) could help link biological variation to brain function in regions critical for impulse regulation, such as the prefrontal cortex and basal ganglia. However, such multimodal integration poses statistical challenges that require careful modeling strategies, including approaches like partial least squares or structural equation modeling (Smith and Nichols, 2018).

Finally, these findings may have practical applications in personalized coaching or mental skills training, though such uses must be guided by robust ethical safeguards and further validation. At present, no standardized protocols exist for incorporating epigenetic biomarkers into sports training, underscoring the need for caution before translation into applied contexts.

## 5 Conclusion

This study provides evidence that behavioral self-regulation in elite combat sport athletes may be linked to the combined influence of genetic background, epigenetic regulation, and long-term environmental exposure. Athletes exhibited higher *DAT1* promoter methylation and lower impulsivity compared to matched controls, and *DAT1* methylation showed a significant interaction with *DRD2* rs1076560 genotype, suggesting that dopaminergic genetic variability may be associated with experience-dependent epigenetic patterns in high-performance contexts.

Overall, these findings support an integrative biopsychosocial perspective in which training-related demands and biological predispositions jointly contribute to individual differences in self-regulation. By combining molecular and psychological measures, the present work highlights elite combat sports as a valuable model for studying adaptive mechanisms of impulse control beyond clinical frameworks. Longitudinal and multimodal studies are needed to determine whether these epigenetic signatures track training adaptation over time.

## Data Availability

The raw data supporting the conclusions of this article will be made available by the authors, without undue reservation.
